# A wheat protein kinase gene *TaSnRK2.9*-5A associated with yield contributing traits

**DOI:** 10.1007/s00122-018-3247-7

**Published:** 2018-12-05

**Authors:** Shoaib Ur Rehman, Jingyi Wang, Xiaoping Chang, Xueyong Zhang, Xinguo Mao, Ruilian Jing

**Affiliations:** National Key Facility for Crop Gene Resources and Genetic Improvement, Institute of Crop Sciences, Chinese Academy of Agricultural Sciences, Beijing, 100081 China

## Abstract

**Key message:**

We developed breeder-friendly high-throughput and cost-effective KASP marker for marker-assisted selection for grain yield related traits in wheat.

**Abstract:**

Plant-specific protein kinase, SnRK2s, is a major family of signaling genes associated with metabolic regulations, nutrient utilization and response to external stimuli. In the present study, three copies of *TaSnRK2.9* were isolated from chromosomes 5A, 5B and 5D of wheat (*Triticum aestivum* L.). The coding regions of *TaSnRK2.9*-5A, *TaSnRK2.9*-5B and promoter region of *TaSnRK2.9*-5D were investigated for sequence polymorphism. Single nucleotide polymorphisms (SNPs) were identified for *TaSnRK2.9*-5A, while no polymorphism was identified in *TaSnRK2.9*-5B and *TaSnRK2.9*-5D. The nucleotide sequence of *TaSnRK2.9*-5A consisted of 2180 bp having eight introns and nine exons. Three SNPs were identified at 308 nt, 698 nt and 1700 nt. For high-throughput genotyping, two kompetitive allele-specific PCR (KASP) markers were developed. Four haplotypes *Hap*-5A-1, *Hap*-5A-2, *Hap*-5A-3 and *Hap*-5A-4 were detected in wheat populations collected from China, Europe and Pakistan. Association analysis was performed with mixed linear model in TASSEL (v 5.0). The results indicated that *Hap*-5A-1/2 of *TaSnRK2.9*-5A were significantly associated with high thousand kernel weight, while *Hap*-5A-4 with high grains per spike. Overexpressing transgenic rice also showed higher grains per spike which is in accordance with association analysis results. Geographic distribution and allelic frequency indicted that the favored haplotypes were positively selected in Chinese (*Hap*-5A-1/2), Pakistani (*Hap*-5A-1), east European (*Hap*-5A-1) and west European (*Hap*-5A-4) wheat breeding. The results suggest that the developed KASP markers can be utilized in yield improvement by marker-assisted selection in wheat breeding.

**Electronic supplementary material:**

The online version of this article (10.1007/s00122-018-3247-7) contains supplementary material, which is available to authorized users.

## Introduction

Crop productivity is limited by diverse climatic fluctuations including drought, extreme temperatures (cold and heat), and water logging. Breeding for stress resilient crop varieties is vital to sustain productivity of food commodities. A comprehensive understanding of gene networks underlying important adaptive traits showing resilience to weather extremes would benefit breeding for new cultivars (Mickelbart et al. 2015). Common wheat (*Triticum aestivum* L.) is the staple food of more than 4.5 billion people in 94 developing countries (Braun et al. 2010), and doubling the wheat production by 2050 due to population surge in the face of climate change and continuously declining arable land is a big challenge (Li et al. 2018). Therefore, breeding for high grain yield is the utmost objective and there is continuous search for genes underpinning yield and its direct contributing traits.

Wheat grain yield per unit area is the product of grain weight and grain number per unit area (Reynolds et al. 2009; Sreenivasulu and Schnurbusch 2012). Variable correlations between grains per spike (GPS) and thousand kernel weight (TKW) have been reported. In bi-parental populations, significantly negative correlation has been reported between the two traits (Kuchel et al. 2007; Mcintyre et al. 2010; Wu et al. 2012). Whereas, non-significant correlation was also identified for GPS and TKW in French winter wheat collections (Brancourt-Hulmel et al. 2003), CIMMYT-derived spring wheat collection (Maqbool et al. 2010) and Chinese landraces (Zhang et al. 2012a). However, a significant positive correlation has also been reported between GPS and TKW in Chinese modern cultivars (Zhang et al. 2012a). Several causal genes for grain traits including TKW and grain number have been cloned and their functional markers (FMs) have been developed including *TaSnRK2.3*-*1A/1B* (Miao et al. 2017), *TaSnRK2.10*-*4A* (Zhang et al. 2017), *TaFlo2*-*A1* (Sajjad et al. 2017), *TaTGW6*-*A1* (Hanif et al. 2016), *TaGS5*-*3A* (Ma et al. 2016), *TaCwi*-*A1* (Ma et al. 2012), *TaSus2*-*2B* (Jiang et al. 2011), *TaGW2* (Su et al. 2011), *TaCKX6*-*D1* (Zhang et al. 2012b), *TaSAP1*-*A1* (Chang et al. 2013), *TaGS1a* (Guo et al. 2013), *TaGS*-*D1* (Zhang et al. 2014) and *TaGASR7*-*A1* (Dong et al. 2014).

Gel-based markers may be less suitable for genotyping a few SNPs on tens of thousands of plants, because they are cost-ineffective. In such cases, cost-effective genotyping platform such as Kompetitive Allele-Specific PCR (KASP) will be more effective and suitable for marker-assisted selection. Single-plex KASP assays offer high-throughput genotyping that is appropriate to screen thousands of genotypes in days with low cost per sample, minimum error rates, and quick turnaround.

Reversible phosphorylation of protein is the main theme in cell signaling involved in abiotic and biotic stress adaptation (Lata et al. 2015). At present, several protein kinase families have been reported involved in response to abiotic stresses. Calcium-dependent protein kinase (CDPK) (Ludwig et al. 2003), mitogen activated protein kinase (MAPK) (Wrzaczek and Hirt 2001) and sucrose non-fermenting 1 (SNF1)-related protein kinase (SnRK) (Hardie 2007) are the main stress-inducible protein kinases families. Recently, SnRK2s attracted much more attention because of its roles in seed dormancy, development and various environmental signaling (Coello et al. 2011).

On the basis of sequence similarity, expression pattern and gene structure, SnRK family is grouped into three sub-families in plants, i.e., SnRK1, SnRK2 and SnRK3 (Hrabak et al. 2003). SnRK1 plays crucial role in sucrose signaling and carbon metabolism in plants, while SnRK2 and SnRK3 are involved in responses to abiotic stresses (Halford and Hey 2009). SnRK2 are plant-specific canonical serine/threonine protein kinases, having N-terminal catalytic domain involved in kinase activation and a regulatory C-terminal domain involved in protein–protein interaction and possibly in ABA and signaling (Vlad et al. 2009). The C-terminal domain is relatively shorter and is abundant in Asp/Glu and is predicted to be coiled-coil (Vlad et al. 2009). According to varied activation patterns in response to ABA, and protein structure, SnRK2s are further divided into three sub-classes, namely subclass I, II and III, having no, weak and strong response to ABA, respectively (Kulik et al. 2011).

To date, SnRK2s are mainly reported for their involvement in response to abiotic stresses (Zhang et al. 2011a). Increasing evidence reveals that SnRK2s have also acquired various regulatory properties in growth and developmental processes in crop plants (Boudsocq et al. 2004; Mao et al. 2010). Gene mapping results showed that *TaSnRK2.7*-*B* was co-located in the same or adjacent chromosome intervals with QTLs for phosphorus utilization efficiency and accumulation efficiency of stem water-soluble carbohydrates (Zhang et al. 2011a, b), and *TaSnRK2.3* co-located with a quantitative trait locus controlling total root length and plant height (Tian et al. 2013). Association analyses have confirmed that *TaSnRK2.3*-1A and *TaSnRK2.3*-1B, as well as the haplotypes of *TaSnRK2.10*-4A involved in regulation of TKW (Miao et al. 2017; Wang et al. 2014). Previous findings related to gene expression analysis from our group suggested that *TaSnRK2.9* was highly sensitive to osmotic stress because this stressor strongly induced transcription within 1 h of exposure and generally caused highest level of transcription compared to other stressors (Zhang et al. 2016). In this study, *TaSnRK2.9s* were isolated and their sequence polymorphisms were assayed for developing functional markers. Breeder-friendly high-throughput KASP markers were developed. Association analysis was performed to reveal the association of haplotypes with yield-related traits. Geographic distribution of haplotypes was also investigated among wheat cultivars and landraces from China, Pakistan and Europe.

## Materials and methods

### Wheat plant material and phenotyping

A drought tolerant cultivar “Hanxuan 10” was used to isolate the coding and promoter sequence of *TaSnRK2.9*. A panel of 34 highly diverse wheat accessions was used to identify DNA sequence polymorphism of target genes. Three natural populations (NP) of common wheat were used for association analysis. Natural population 1 (NP-1) consisted of 323 winter wheat accessions, of which 318 were collected from China (12 landraces, 36 advanced lines, and 270 modern cultivars), mainly under cultivation in Yellow and Huai River Valleys Facultative Wheat Zone and Northern Winter Wheat Zone. The remaining 5 accessions were Early Premium, Triumph, Lovrin 10, Salgemma and Drysdale from America, Rumania, Italy and Australia, respectively. Natural population 2 (NP-2) comprised of 157 landraces, and natural population 3 (NP-3) consisted of 348 modern cultivars. Both NP-2 and NP-3 came from Chinese wheat core collection (Zhang et al. 2002). The NP-2 represents more than 70% of the total genetic diversity of the Chinese wheat germplasm collection (Hao et al. 2008, 2010). The NP-3 was used to identify progressive haplotypes and geographic distributions of favored and unfavored haplotypes of *TaSnRK2.9*-5A. In addition, 75 Pakistani and 313 European wheat cultivars were also surveyed to investigate the geographic distribution of *TaSnRK2.9*-5A haplotypes in these two regions.

NP-1 was planted in 10 different environments (year × site × water regime × heat stress) in Changping (40°13ʹN; 116°13ʹE), Beijing, in 2014–2015 and 2015–2016, and Shunyi (40°23ʹN; 116°56ʹE), Beijing, 2015–2016. Field experiments were carried out under drought-stressed (D), drought-heat-stressed (DH), well-watered (WW), and well-irrigated heat-stressed (WH) experimental units. Thermal stress shelters were built at flowering stage by covering plastic film on steel frames over the trail plots, which was installed before sowing. During the heat stress period, the average highest temperature outside the shelters was 35 °C; the average high temperature inside the shelters under drought stress condition was 45 °C, whereas in the shelters with WW conditions the temperature was 42 °C. The “D” experimental units were rain-fed. The rainfalls in the two growing seasons were 161 mm and 173 mm, respectively. And precipitations mainly occurred in May and June at the experimental stations. Water contents of different soil profiles were measured at booting and grain-filling stages for each growth season (Table S1). The “WW” experimental units were irrigated with 750 m^3^ ha^−1^ (75 mm) at each of pre-overwintering, booting, flowering and grain-filling stages when the amounts of rainfall were insufficient during each corresponding period. Each experimental plot was 2 m length with 4 rows and row spacing of 30 cm, 40 seeds per row. The wheat accessions were sown in the beginning of October, and harvested in the middle of June of the following year. NP-1 was planted for measurement of agronomic traits, such as plant height (PH), spikes per plant (SPP), spikelet number per plant (SNPS), GPS, TKW and yield per plant (YPP).

### Cloning *TaSnRK2.9* in wheat and generation of transgenic rice plant material

*OsSAPK9* cDNA sequence was blasted in wheat EST database. A consensus contig was obtained and a pair of primers (cDNA F/R) was designed to amplify wheat cDNA induced by PEG-treatment (Two-leaf hydroponic wheat seedlings of Hanxuan 10 were exposed to − 0.5 MPa PEG-6000) (Table 1). *TaSnRK2.9* cDNA was amplified by using *pfu* DNA polymerase, and the target fragments were then ligated with *pEASY*-Blunt cloning vector. After sequencing, two endonuclease enzyme cutting sites for *Bam*HI and *Spe*I, were added to upstream and downstream of the ORF, respectively, and a 30 bp MYC-tag sequence was inserted between *Bam*HI site and the first ATG of *TaSnRK2.9*, followed by sub-cloning into binary vector pCAMBIA1391 cut with corresponding enzymes.

**Table 1:** Primers used in the experiments

Primer name	Primer sequence 5′ to 3′	Purpose
cDNA-F/R	F: GCGAGCGAGAGAGATAAGG R: ACTCAAGTAGTAGCCTGAATAC	cDNA amplification
Sub-cloning primers	F: CT*ggtacc*ATGGAGCAGAAACTCATCTCTGAAGAGGATATGGAGAGGGGGCCGG R: CT*actagt*CTACATGGCGTATACTATCTCCCCG	Sub-cloning in binary vector
qRT-PCR-F/R	F: CGACGAGGCTCGCTTCTTTT R: CCCACAGTTGACTTTGGTTGA	Gene expression level detection in transgenic rice
Tubulin	F: TGAGGACTGGTGCTTACCGC R: GCACCATCAAACCTCAGGGA	Internal control qRT-PCR primers
AB-F/R	F: GCGGGGATCTCCGTGTC R: TGCACATACAGATATTCACAGGTT	Genomic fragment amplification
D-F/R	F: GGAGAAGAGGCACCAAGAACAG R: CGCCCTCTCTCCTTATCTCTC	D promoter region amplification
Seq-A-F1 Seq-D-F1	F: CGACGCATCTCGCCATC F: GCTGAACTTAAAAGCCCCC	Sequencing primers for *TaSnRK2.9*
M13	F: TGTAAAACGACGGCCAGT R: CAGGAAACAGCTATGACC	
Ta5A1-F/R	F: GCTGAGTGATGTGCCGGTG R: TCGTAGTAATTTTCACTCACCTCCTT	Chromosomal location check
KASP1	F: GAAGGTGACCAAGTTCATGCTCTTGGCACCAGACCAGAGCCACGGC F: GAAGGTCGGAGTCAACGGATTCTTGGCACCAGACCAGAGCCACGGT R: ACGCATCATCAAACTTGTAAATACC	KASP assay for SNP at 308 nt (C/T)
KASP2	R: GAAGGTGACCAAGTTCATGCTTGAATGTAGTCCGGAATCGAGTACG R: GAAGGTCGGAGTCAACGGATTTGAATGTAGTCCGGAATCGAGTACT F: CCGAGCTCAACTTTTTCAGAAAA	KASP assay for SNP at 1700 nt (A/C)

Small caps italic letters show *Bam*HI (*ggtacc*) and *Spe*I (*actagt*) sites. Underlined letters show 30-bp MYC-tag sequence

After the transformation of target gene into Agrobacteria EHA105, the construct was transferred into wild-type (WT) rice (Kittakee) by Agrobacteria-mediated method. Positive transgenic plants were initially screened by PCR, and then reconfirmed by sequencing. T3 pure lines were used for phenotyping analysis, and the expression level of target gene was identified by qRT-PCR, in triplicate with Roche LightCycler 96-well Real-Time PCR system (Roche, Switzerland) using SYBR Green PCR Master Mix Kit (Takara, Japan). Rice *Tubulin* was used as an internal control to quantify the relative transcript level of wheat target genes (Table 1). Thermal cycling conditions were pre-incubated at 95 °C for 2 min, followed by 95 °C for 10 s, 60 °C for 30 s, and 72 °C for 30 s for 45 cycles. The relative transcription level for each gene was calculated using 2^−ΔΔCT^ method (Livak and Schmittgen 2001) (Table 1). WT and transgenic rice lines were planted at the Institute of Crop Sciences, Experimental Station in Beijing (39°48ʹN; 116°28ʹE). Rice seeds were germinated in water for 4 days followed by transplantation in plastic containers (Length × width × height = 80 × 35 × 30 cm) in the middle of June 2017. This experiment was performed in randomized complete block design with three replications. The agronomic traits including plant height (PH), spike length (SPL), number of fertile tillers (NFT), GPS and TKW were measured at maturity. To measure agronomical traits of transgenic rice, 27 plants were sowed in uniform tank containing the same amount of soil, and the management practices for WT and transgenic lines was same.

### Chromosomal location

To identify the chromosomal location of target genes, the genomic sequences were used as queries to do BLAST online (https://urgi.versailles.inra.fr/blast/blast.php). Chromosomal location was further tested by PCR amplification in diploid, tetraploid, hexaploid wheat species and nulli-tetrasomic and ditelosomic lines of Chinese Spring using Ta5A1-F/R primers.

### Isolation and sequencing of *TaSnRK2.9*

DNA of 34 highly polymorphic wheat accessions (Li et al. 2016) was extracted from young leaves with CTAB method (Stewart and Via 1993). A pair of primers (AB-F/R) was selected to amplify the genomic sequence of *TaSnRK2.9* in A and B genomes of wheat. A pair of primers (D-F/R) was also designed to amplify the promoter region of *TaSnRK2.9* in D genome (Table 1). TransStart Fast *Pfu* DNA polymerase: LA-Taq = 1:1 was used for PCR amplification. PCR was performed in a total volume of 20 µL having 4 µL 5 × *Pfu* buffer, 0.4 µL *Pfu *+ LA-Taq mixture, 0.2 µL dNTP (25 mM for each nucleotide), 1.2/1.2 µL forward and reverse primer (10 µM), 2.2 µL DNA (100 ng µL^−1^), and 10.8 µL ddH_2_O. PCR conditions were 95 °C for 5 min; 34 cycles of 95 °C for 1 min, 59 °C for 40 s, 72 °C for 3 min; followed by a final extension of 72 °C for 10 min. PCR product was checked on agarose gel (1.2%), and desired band was isolated and extracted using BioTeke DNA extraction kit, followed by cloning into *pEASY*-Blunt vector. Twenty-four positive clones for each sample were selected for sequencing by DNA Analyzer 3730XL. To get the full-length desired sequence of *TaSnRK2.9*-5A and *TaSnRK2.9*-*5B,* M13 forward and reverse primers and an overlapping sequencing primer (Seq-A-F1) were used for sequence walking. For D genome promoter region sequencing, ten positive clones were sequenced with M13 forward and reverse and an overlapping sequencing primer (Seq-D-F1) (Table 1). The sequence of each clone was obtained by assembling with SeqMan program in DNAStar software package. The genomic origin of each sequence was firstly confirmed by comparing with reference genomic sequence obtained from URGI (https://urgi.versailles.inra.fr/blast/blast.php).

### Functional marker development

For high-throughput genotyping, KASP primers were developed on the two SNP sites (KASP1 at 308 nt and KASP2 at 1700 nt in A genome) by following standard KASP guidelines (http://www.lgcgenomics.com. The allele-specific primers were developed having the standard FAM (5′ GAAGGTGACCAAGTTCATGCT 3′) and HEX (5′ GAAGGTCGGAGTCAACGGATT 3′) tails with a targeted SNP at the 3′ end (Table 1). For KASP1, two forward primers (allele specific) and one common reverse primer was designed. However for KASP2, two reverse primers (allele specific) and one common forward primer was designed. A common primer was designed so that the total fragment length was less than 120 bp. KASP makers were then applied across the entire NP-1, NP-2, NP-3, Pakistani and European wheat accessions. The PCR mixture consisted 30 µL common primer (100 µM), 12 µL of each tailed primer (100 µM), and 46 µL ddH_2_O. Assays were tested in ~ 4 µL reaction mixture (50 ng/µL DNA, 2.5 µL of 1 × KASP master mixture, 0.04 µL MgCl_2_, 0.056 µL primer mixture (all three primers), and 0.205 µL ddH_2_O. PCR conditions were as: hot start at 95 °C for 15 min, followed by 10 touch down cycles (95 °C for 20 s; touch down at 65 °C initially and decreasing by − 1 °C per cycle for 25 s), followed by 30 more cycles of annealing (95 °C for 10 s; 57 °C for 60 s). An extension step did not performed as the fragment lengths were less than 120 bp. Fluorescence levels were detected by Synergy H1MF Microplate Reader (BioTek Instruments, Inc, USA) by following the manufacturer’s guide and analyzed using KLUSTERCALLER software (version 3.4.1.36; LGC Hoddesdon, UK).

### Association analysis

Descriptive statistics and estimates of variance were conducted using Microsoft Excel 2013. TASSEL (v 5.0) was used to identify significant associations between agronomic traits and haplotypes for NP-1. Mixed linear model (MLM) was executed using population structure (Q) and kinship (K) matrix to control spurious associations. Associations were considered statistically significant at *P *< 0.05. The effects of haplotype on each trait were also analyzed by *Student’s t test* at *P *< 0.05 (even 0.01).

## Results

### Genetic characterization of *TaSnRK2.9*

BLAST results showed that *TaSnRK2.9* was located on chromosome 5A, 5B and 5D, and were named as *TaSnRK2.9*-*5A*, *TaSnRK2.9*-*5B* and *TaSnRK2.9*-*5D*, accordingly. Gene structure showed that *TaSnRK2.9* was consisted of 9 exons and 8 introns. Additionally, the promoter region for *TaSnRK2.9*-*5D* gene was also isolated, and named as *TaSnRK2.9*-*5D*-*PRO.* The fragment sizes of *TaSnRK2.9*-*5A, TaSnRK2.9*-*5B,* and *TaSnRK2.9*-*5D*-*PRO* were 2180, 2137, and 2200 bp, respectively.

**Fig. 1:**
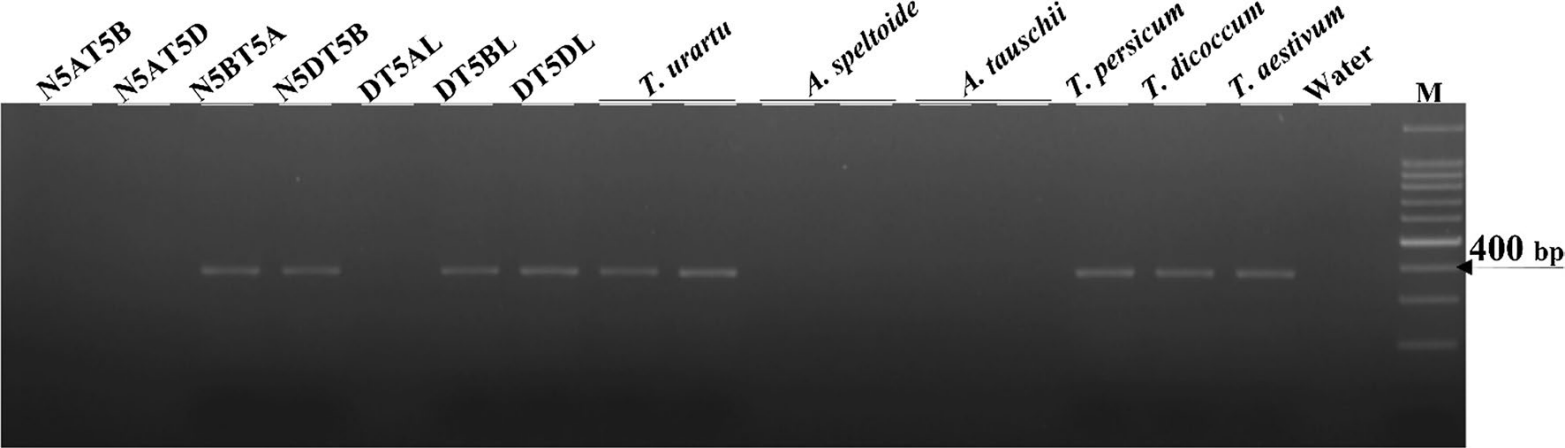
Chromosomal location of *TaSnRK2.9*-5A. *TaSnRK2.9*-5A was located on chromosome 5A using nulli-tetrasomic and ditelosomic (DT) lines of Chinese Spring. M, DNA100 bp marker. *T. urartu* (AA), *A. speltoide* (BB), *A. tauschii* (DD), *T. persicum* (AABB), *T. dicoccum* (AABB), *T. aestivum* (AABBDD)

To further determine the chromosomal location of *TaSnRK2.9*, a pair of genome specific primer (Ta5A1-F/R) for *TaSnRK2.9*-5A was designed. The amplified fragment was 380 bp in length. As shown in Fig. 1, the amplified fragment was identified only in species carrying “A” genome. This was further confirmed by PCR amplification in nulli-tetrasomic and ditelosomic lines of Chinese Spring, revealing that *TaSnRK2.9*-5A was located on the short arm of chromosome 5A (Fig. 1).

### Sequence polymorphism assays and marker development

**Fig. 2:**
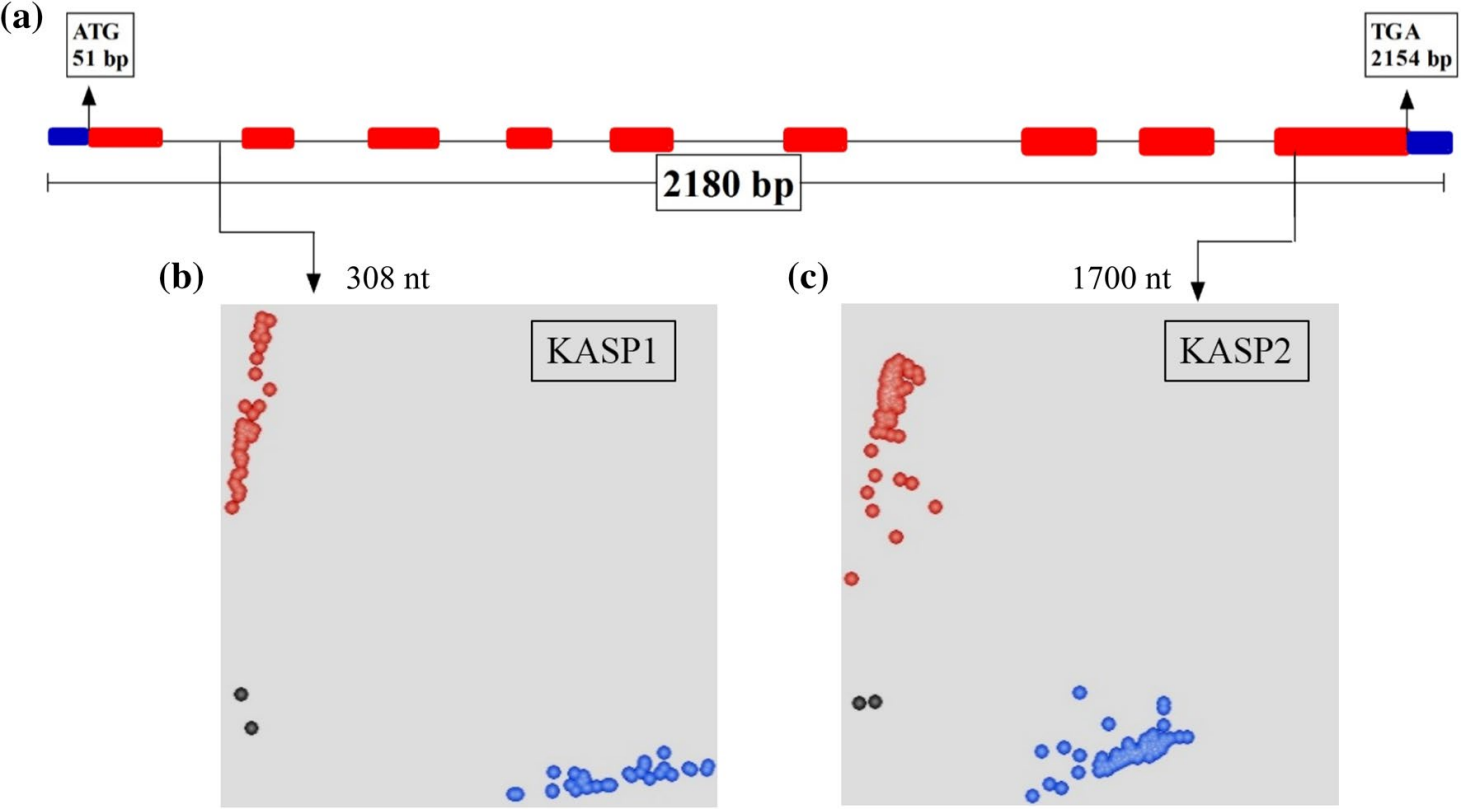
Gene structure and functional marker development for *TaSnRK2.9*-5. **a** Gene structure, blue blocks = untranslated region, red blocks = exon, black line = intron, M = DNA marker (100 bp). Scatter plot for KASP assays; showing clustering of accessions on the *X*—(FAM) and *Y*—(HEX) axes. Accessions colored blue have the FAM-type allele; accessions colored red have the HEX-type allele; black dots represent the NTC (non-template control). **b** KASP1 assay for SNP-308 nt shows allele “C” in FAM and allele “T” in HEX cluster. **c** KASP2 assay for SNP-1700 nt shows allele “C” in FAM and allele “A” in HEX cluster

For *TaSnRK2.9*-*5B* coding and *TaSnRK2.9*-*5D* promoter region, no polymorphism was identified, hence were excluded from further analysis. For *TaSnRK2.9*-5A, polymorphic sites were identified in both intron and exon regions (Fig. 2a). The SNP at 308 nt (C/T) was identified in intron and caused no amino acid change. The SNP at 1700 nt (A/C) in exon led to an amino acid change (CAG → Gln to CCG → Pro) without changing protein 3D structure (Supplementary Fig. 1). KASP markers were developed at the both SNP sites. KASP1 was developed for SNP at 308 nt, whereas KASP2 was developed for SNP at 1700 nt (Fig. 2b, c). Scatter plot for KASP assay shows clustering of accessions on the *X*—(FAM) and *Y*—(HEX) axes. Strong FAM signal results in clogging of blue dots (genotypes) at extreme right bottom while strong HEX signal results in clogging the red dots (genotypes) at extreme top left corner of the figure. For KASP1, accessions colored blue have “C” allele, while accessions colored red have “T” allele. For KASP2, accessions colored blue have “C” allele, whereas accessions colored red have “A” allele.

### Association analysis of *TaSnRK2.9*-5A haplotypes and agronomic traits

**Fig. 3:**
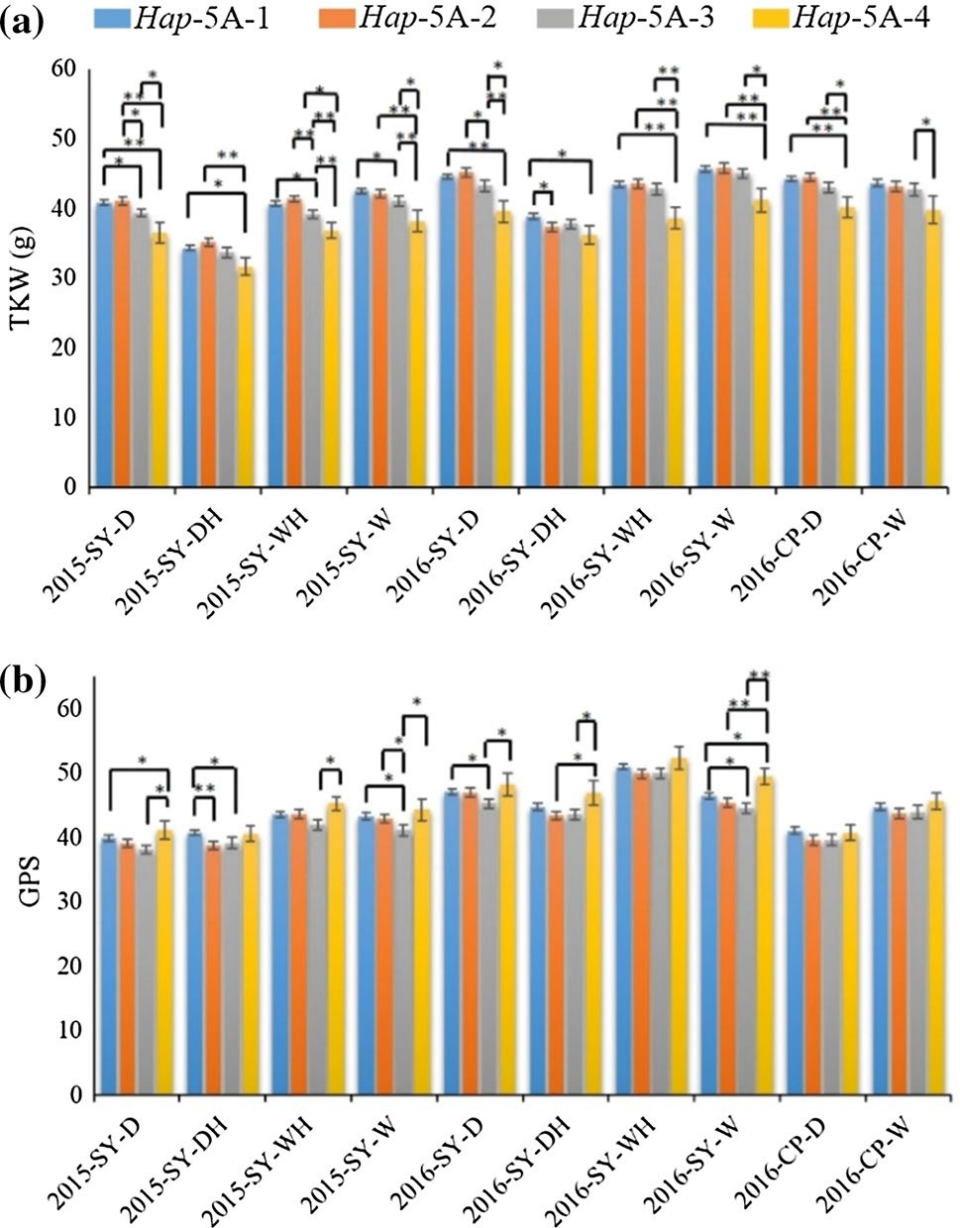
Phenotypic comparison of four *TaSnRK2.9*-5A haplotypes in ten environments in NP-1. **a** Traits are thousand kernel weight—TKW. **b** grains per spike—GPS. *P < 0.05, **P < 0.01. Error bars denote SE

Four haplotypes of *TaSnRK2.9*-5A, i.e., *Hap*-5A-1 (TA), *Hap*-5A-2 (TC)*, Hap*-5A-3 (CC) and *Hap*-5A-4 (CA) were identified in wheat population NP-1. *Hap*-5A-1 was the most frequent haplotype available in 54.8% accessions of NP-1, followed by *Hap*-5A-2 (22.5%), *Hap*-5A-3 (17.3) and *Hap*-5A-4 (5.2%). Association analysis showed that at unique field sites, *TaSnRK2.9*-5A was associated with TKW in four out of ten and GPS six out of ten environments (Table 2). Both *Hap*-5A-1 and *Hap*-5A-2 (*Hap*-5A-1/2) were favored haplotypes for higher TKW relative to *Hap*-5A-3/4 (Fig. 3a). *Hap*-5A-4 had significantly higher GPS compared to other haplotypes (Fig. 3b).

**Table 2:** Association of *TaSnRK2.9*-5A haplotypes with agronomic traits in individual environments in NP-1

Environments	TKW (*P* value)	GPS (*P* value)	SPP (*P* value)	SNPS (*P* value)
2015-SY-D	0.039*	NS	0.016*	NS
2015-SY-DH	NS	NS	NS	NS
2015-SY-WH	0.011*	0.012*	0.019*	NS
2015-SY-W	NS	9.38E−04***	NS	NS
2016-SY-D	0.044*	0.001**	0.009**	0.001**
2016-SY-DH	NS	0.032*	NS	NS
2016-SY-WH	NS	NS	NS	NS
2016-SY-W	NS	0.019*	NS	0.003**
2016-CP-D	0.043*	0.040*	NS	NS
2016-CP-W	NS	NS	NS	NS

*TKW* thousand kernel weight, *GPS* grains per spike, *SPP* spikes per plant, *SNPS* spikelet numbers per spike, *NS* non-significant. *P < 0.05, **P < 0.01, ***P < 0.001. The ten environments were at Shunyi (SY) and Changping (CP) under drought (D), drought + heat (DH), well-watered (W) and well-watered + heat (WH) conditions in 2015 and 2016

### Phenotyping of *TaSnRK2.9*-5A overexpressing rice

**Fig. 4:**
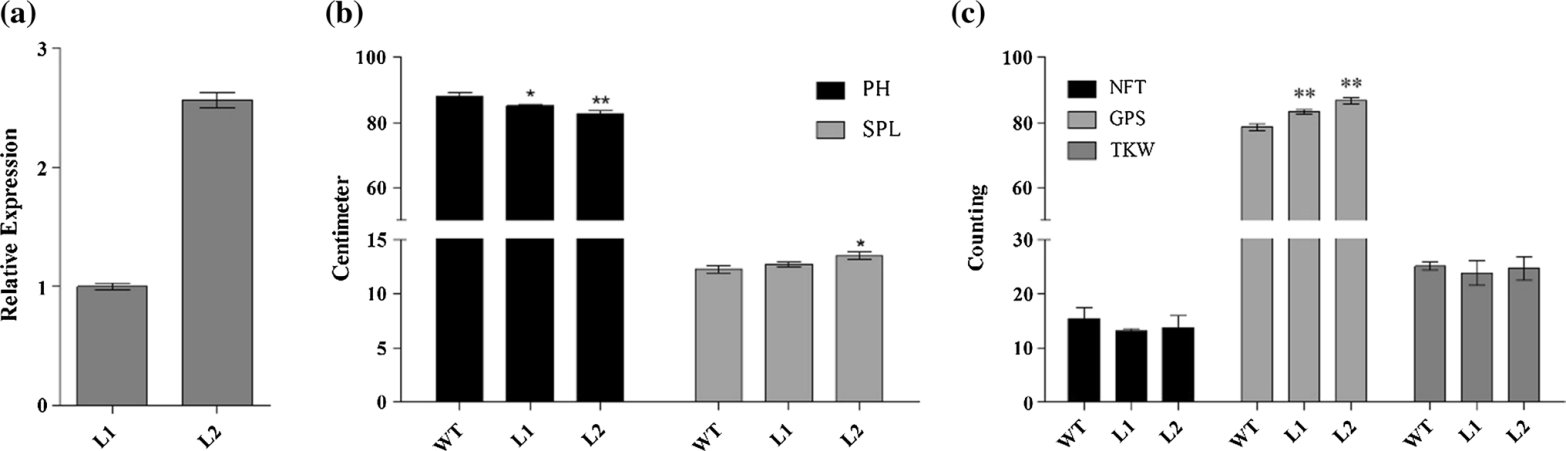
Phenotypes of rice plants overexpressing *TaSnRK2.9*-5A. **a** Relative gene expression of transgenic rice. Comparison of traits in transgenic and control plants for (**b**), plant height (PH) and spike length (SPL), and **c** number of fertile tillers (NFT), grains per spike (GPS) and thousand kernel weight (TKW). WT, wild type; L1, L2, transgenic lines. Error bars denote SE. *P < 0.05, **P < 0.01

Two rice transgenic lines for *TaSnRK2.9*-5A, with higher normalized fold expression (Fig. 4a), were used for phenotyping. Data from six plants of each transgenic rice line and WT were averaged. Although both transgenic lines showed a significantly shorter PH, but significant increments were observed for SPL and GPS compared with WT plants. As compared to WT, transgenic lines showed a significant reduction in PH from 88.0 to 82.8 cm. Spike length increased from 12.3 to 13.5 cm, and GPS increased from 78 to 87. TKW showed slight reduction from 24.8 to 23.9 g (Fig. 4b, c).

### Geographic distribution of *TaSnRK2.9*-5A haplotypes in China, Pakistan and Europe

**Fig. 5:**
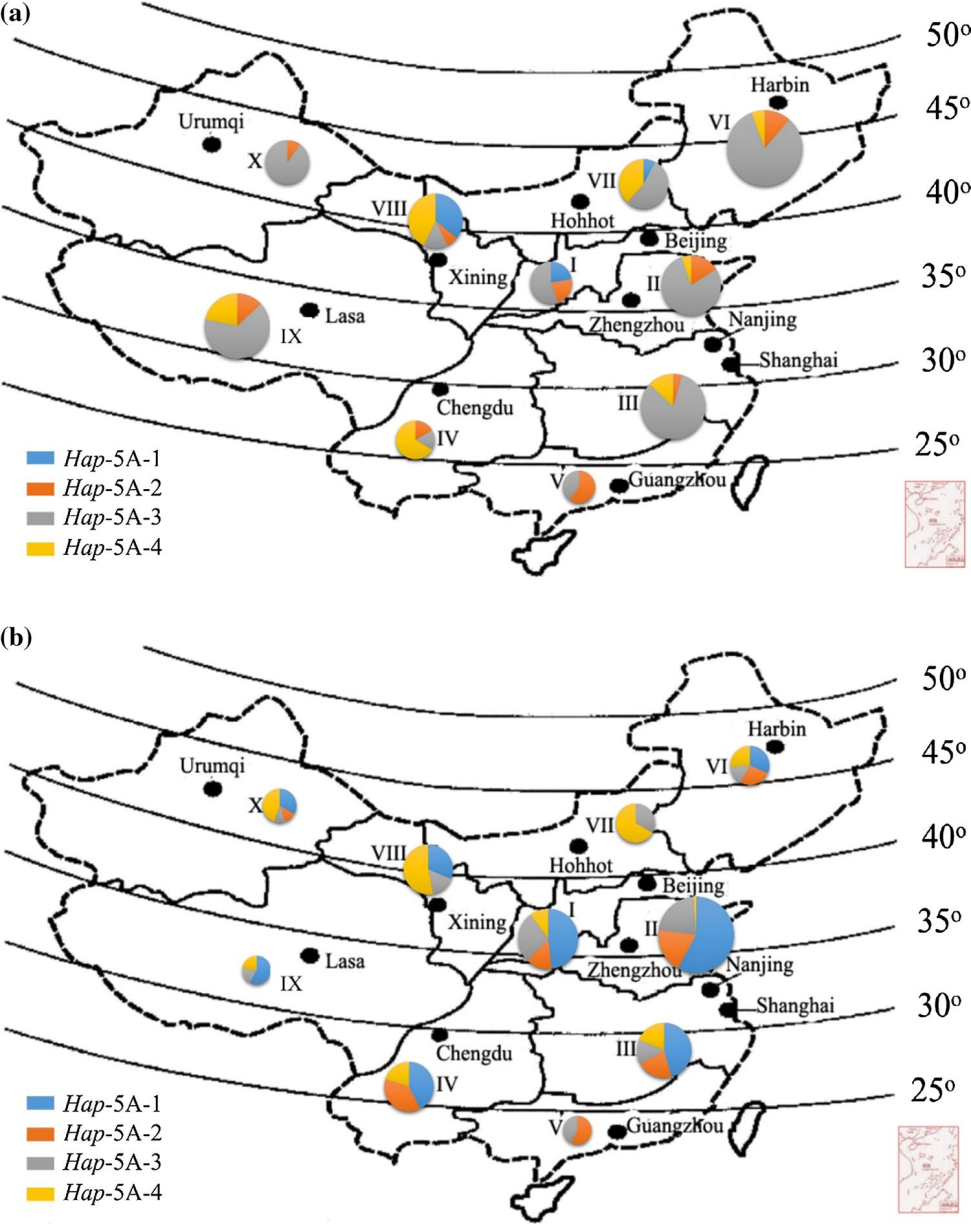
Haplotype distribution of *TaSnRK2.9*-5A in China. **a** Chinese landraces. **b** modern cultivars in 10 major wheat production zones. I, Northern Winter Wheat Zone; II, Yellow and Huai River Valleys Cultivated Wheat Zone; III, Middle and Low Yangtze Valleys Autumn-Sown Spring Wheat Zone; IV, Southwestern Autumn-Sown Spring Wheat Zone; V, Southern Autumn-Sown Spring Wheat Zone; VI, Northeastern Spring Wheat Zone; VII, Northern Spring Wheat Zone; VIII, Northwestern Spring Wheat Zone; IX, Qinghai–Tibetan Plateau Spring–Winter Wheat Zone; X, Xinjiag Winter–Spring Wheat Zone. Size of pie chart is directly proportional to the number of accessions

Previous reports have revealed that the favored haplotypes gradually pyramid due to the crop improvement efforts (Barrero et al. 2011). To determine, whether the favored haplotypes for *TaSnRK2.9*-5A were selected in wheat breeding, we investigated the geographic distribution of *TaSnRK2.9*-5A in NP-2, NP-3, Pakistani and European wheat cultivars. China has three major wheat growing regions which were further divided into 10 agro-ecological zones (He et al. 2001). Zones I–IV are the major wheat producing zones based on production and cultivation area. In NP-2 (landraces), the frequency of the favored haplotypes *Hap*-5A-1/2 for TKW was low, and *Hap*-5A-3 was the pre-dominant haplotype in all major areas except three zones (Zone IV, V, and VIII) (Fig. 5a), suggesting that the selection pressure on haplotypes in different areas was not as strong as anticipated. In NP-3 (modern cultivars), the combined frequency of the two favored haplotypes (*Hap*-5A-1 and *Hap*-5A-2) was higher in the four major zones, i.e., Zones I–IV (Fig. 5b). The frequency of *Hap*-5A-1 increased remarkably from 22 to 48% in Zone I, 0 to 53% in Zone II, 0 to 45.8% in Zone III, and 0 to 42.5% in Zone IV from landraces to modern cultivars, respectively, while the frequency of *Hap*-5A-2 was reduced in Zone I from 22 to 14%, and increased from 15.7 to 17.3% in Zone II, 4.3 to 20.8% in Zone III and 16 to 37.5% in Zone IV from landraces to modern cultivars, respectively. The results indicated that favored haplotypes experienced positive selection in Chinese wheat breeding programs.

**Fig. 6:**
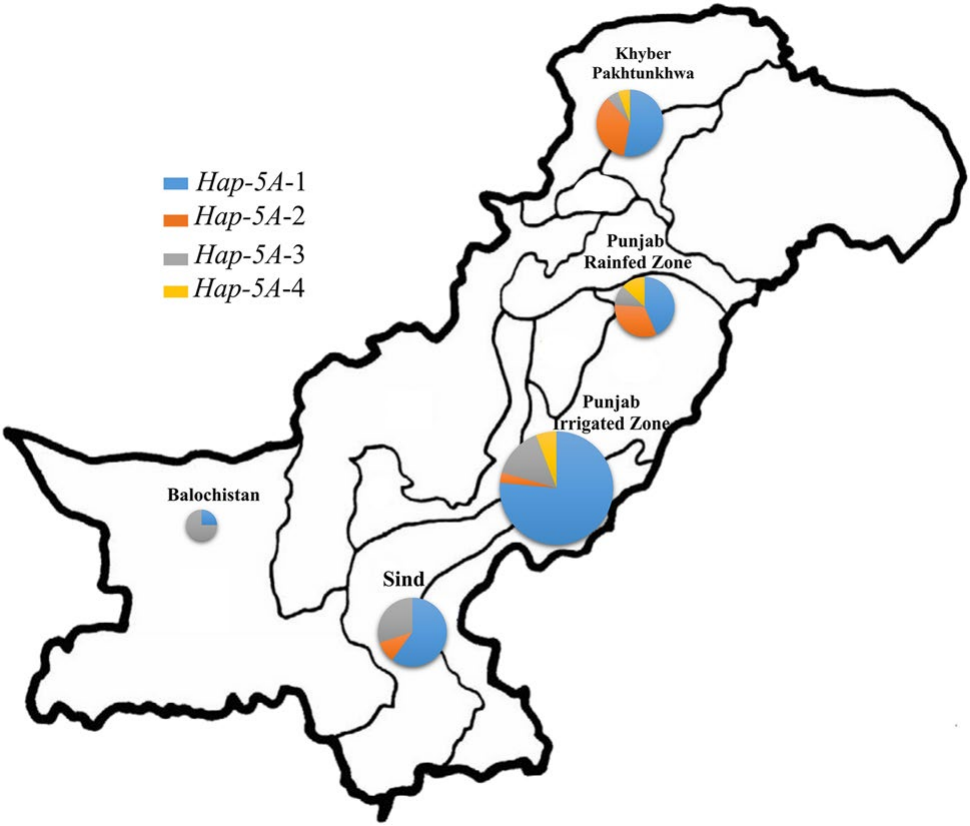
Distribution of *TaSnRK2.9*-5A haplotypes among Pakistani wheat accessions. Size of pie chart is directly proportional to the number of accessions

**Fig. 7:**
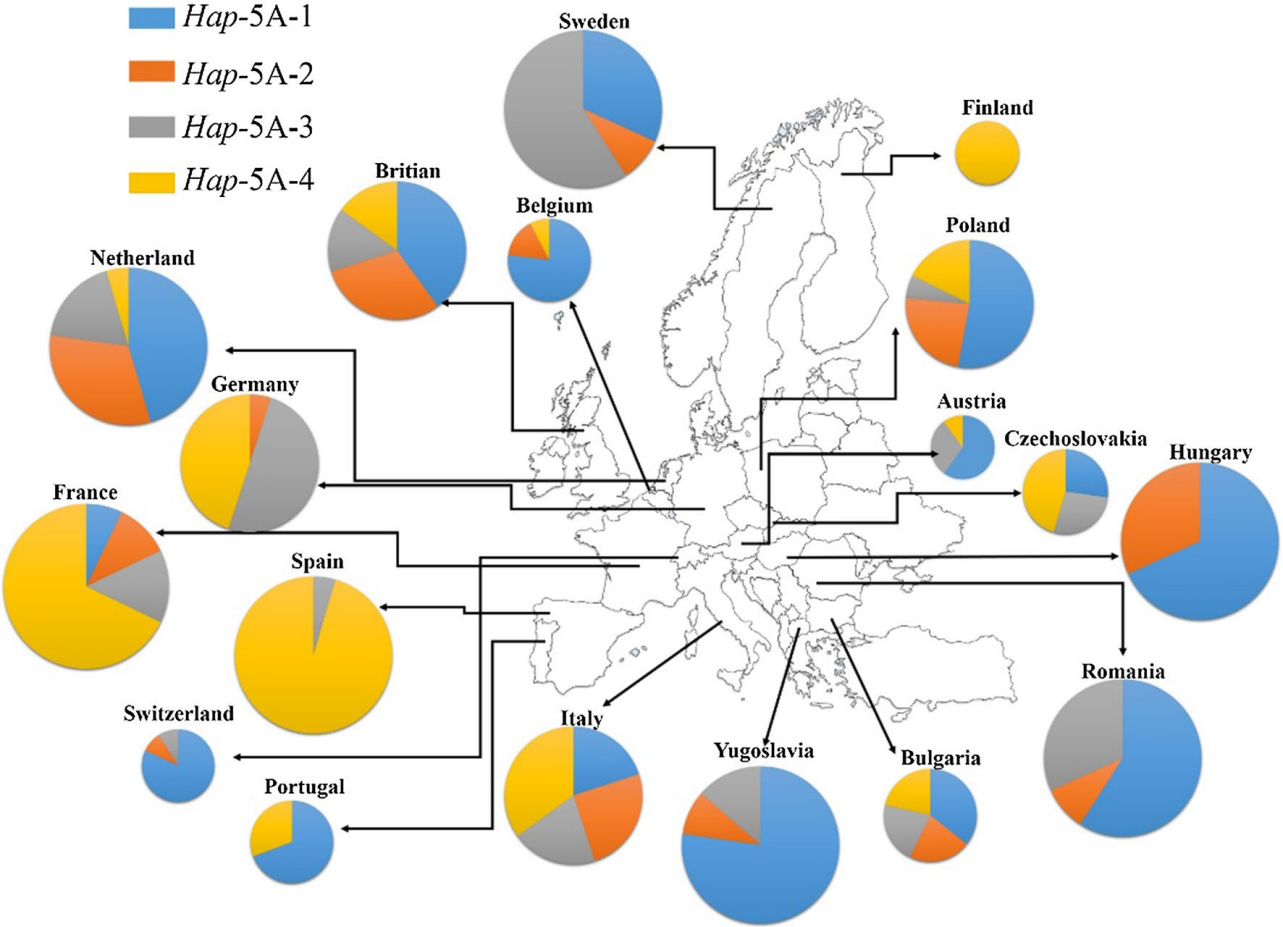
Distribution of *TaSnRK2.9*-5A haplotypes among European wheat accessions. Size of pie chart is directly proportional to the number of accessions

**Fig. 8:**
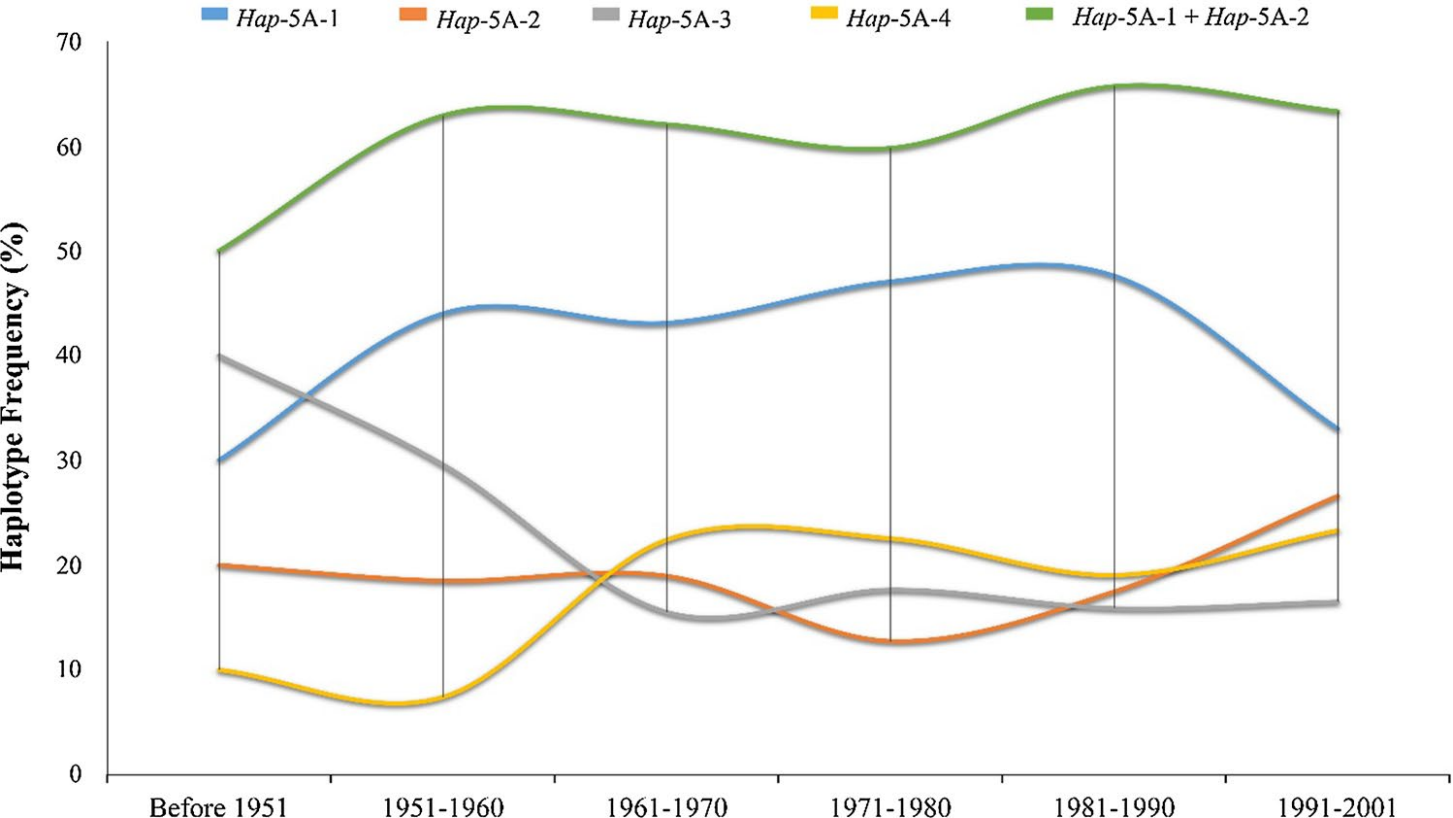
Favored haplotypes were selected in Chinese wheat breeding (NP-3)

Geographic distribution of *TaSnRK2.9*-5A haplotypes was also investigated among Pakistani and European wheat accessions. The combined frequency of *Hap*-5A-1/2 among five different regions of Pakistan was 79.41% (Punjab irrigated zone), 77.77% (Punjab rain-fed zone), 70% (Sind), 88.23% (Khyber Pakhtunkhwa) and 25% (Balochistan) (Fig. 6). The frequencies of favored haplotypes for TKW and GPS were also higher among European wheat accessions (Fig. 7). The combined results demonstrated that *Hap*-5A-1/2 were the dominant haplotypes of *TaSnRK2.9*-5A in China and Pakistan, while *Hap*-5A-1/4 were the prominent haplotypes among European wheat cultivars and selection pressure on these haplotypes differed in degree among these regions.

**Fig. 9:**
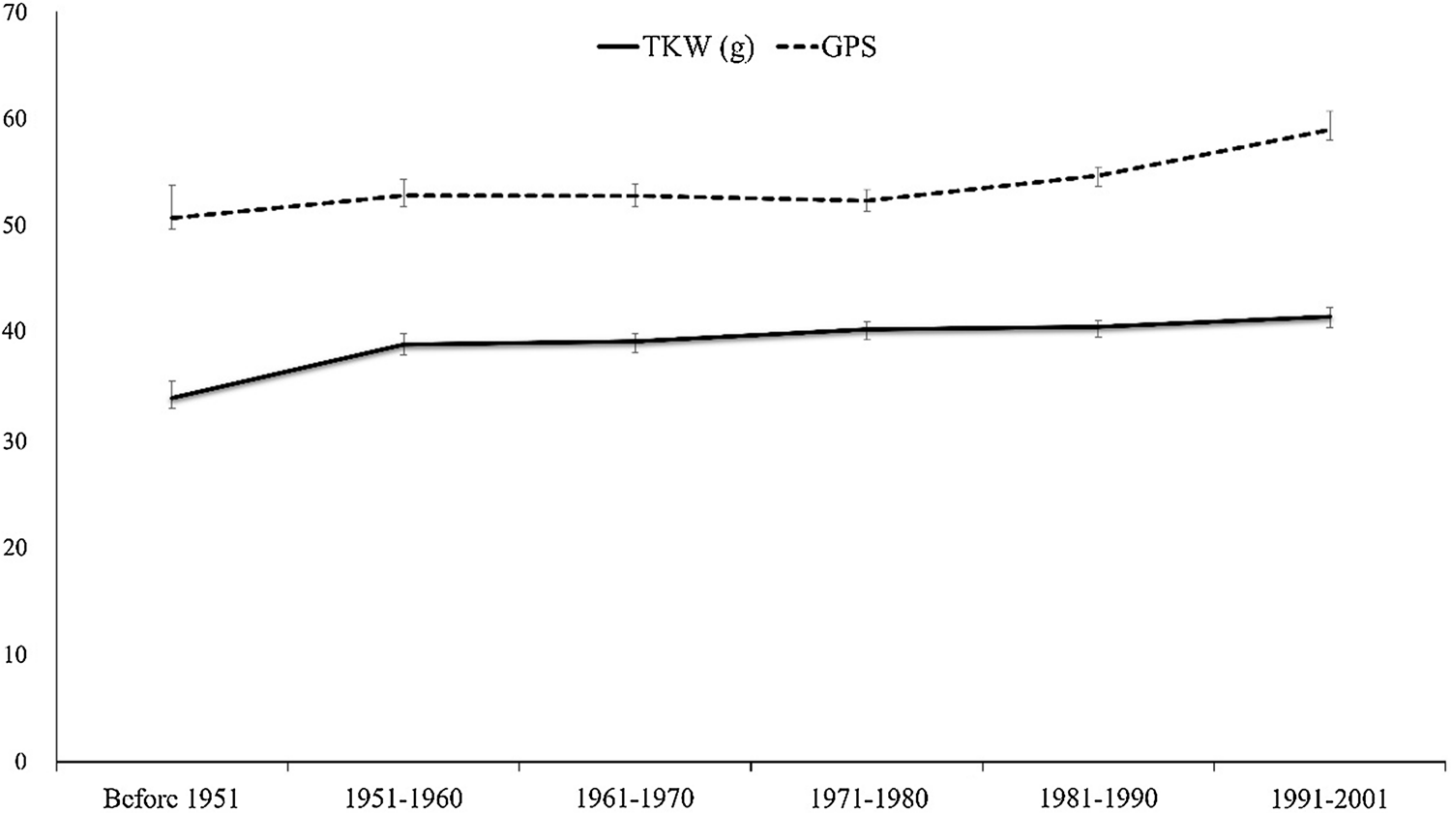
TKW and GPS changes in NP-3 over decades. Error bar indicates SE

### Positive selection of *Hap*-5A-1, *Hap*-5A-2 and *Hap*-5A-4 in China, Pakistan and European wheat breeding process

On the basis of released time, NP-3 was subdivided into six groups, i.e., cultivars released before 1951, 1951–1960, 1961–1970, 1971–1980, 1981–1990 and 1991–2001. The frequency of haplotype *Hap*-5A-3 decreased from 40% before 1951–16.5% in 1991–2001 era (Fig. 8). The frequency of *Hap*-5A-4 increased from 10% before 1951 to 23.3% in 1991–2001 (Fig. 8). The combined frequency of *Hap*-5A-1 and *Hap*-5A-2 continuously increased. From 1971 to onwards, the increase in combined frequency for *Hap*-5A-1/2 revealed a progressive selection (Fig. 8). In Pakistani accessions, a higher frequency for *Hap*-5A-1 was observed (Supplementary Fig. 2). The European population was subdivided into 11 groups according to released date, and the combined frequency of *Hap*-5A-1/2 remained higher followed by *Hap*-5A-4 (Supplementary Fig. 3).

## Discussion

Plant breeding merely through phenotypic selection is relatively ineffective (Gedye et al. 2012), and efficient selections using molecular markers will accelerate the breeding process (Rasheed et al. 2017). Common wheat is an allohexaploid with a large genome size (~ 17 Gb) (Gupta et al. 2008) having polyploid nature and highly similar homologous genes. Genomic studies in wheat were reliant to some extent on comparative genomics approaches with other members of grass family courtesy high collinearity and genetic assembly among grass family members (Murat et al. 2014). At present, the release of reference genome assembly (http://www.wheatgenome.org) has changed that, and paid a smooth way for gene cloning in wheat.

Wheat “D” genome has a narrow genetic background, and the level of polymorphism is relatively small when compared to “A” and “B” genomes (Rasheed et al. 2018). Moreover, non-coding regions have relatively higher level of polymorphism as compared to coding region (Nasu et al. 2002). The absence of polymorphism in *TaSnRK2.9*-5B coding and *TaSnRK2.9*-5D promoter region is probably due to the allele fixation during evolution and domestication or low genetic diversity in the panel used for polymorphism. More work is required to investigate these two probabilities in more diverse wheat accessions and progenitor species. For *TaSnRK2.9*-5A, SNP identified at 1700 bp was within the catalytic domain which led to an amino acid change, i.e., Gln237 to Pro237 but without protein structural alteration (Supplementary Fig. 1). Both glutamine and proline contain an α-amino and α-carboxylic group, and carry positive charge, which might be the reason for the unaltered structures of the two proteins.

Genetic gain in wheat is around 0.8–1.0% per year (Ray et al. 2012), for example, in France (Brancourt-Hulmel et al. 2003), Italy (Canevara et al. 1994), UK (Shearman et al. 2005) and China (Zhou et al. 2007a). The genetic gain has largely been achieved by improvements in grain number per square meter with a slight change in individual grain weight (Gaju et al. 2009). Accessions possessing *Hap*-5A-1/2 of *TaSnRK2.9*-5A had the higher TKW, while accessions possessing *Hap*-5A-4 had higher GPS. Yield-related traits of cereal crops are governed by multiple genes and are strongly influenced by abiotic factors (Zhang et al. 2013). Among the reported gene determinants of cereal grain traits, some genes can act constitutively under different conditions, while others function in particular environmental conditions (Dong et al. 2014). In this study, TKW showed significant association in all individual drought-stressed environmental conditions suggesting that the use of *Hap*-5A-1/2 could be instrumental for higher TKW selection in drought-stressed conditions. Moreover, high GPS in overexpressing *TaSnRK2.9*-5A rice lines might also be an attribute of *Hap*-5A-4 of *TaSnRK2.9*-5A. Higher grain yield has been a major objective of wheat breeders around the globe. In Northern China, 1.3% annual genetic gain for TKW has been achieved over last three decades with simultaneous increase in GPS in Yellow and Huai River Valleys Facultative Wheat Zone (Zhou et al. 2007a). Increase in TKW and kernel weight per spike was also reported in Southern China winter wheat region since 1949 (Zhou et al. 2007b).

From landraces to modern cultivars, the proportion of favored haplotypes increased suggesting a positive selection on *TaSnRK2.9*-5A during wheat breeding (Fig. 5a, b). For *Hap*-5A-1/2, the increase was more apparent in the major wheat production areas. For example, Zones I, II, and III which account for about 64.8% of total national wheat area in China and greater turnover of wheat cultivars were also reported from these Zones. Average TKW of varieties especially in Zone II is about 42–43 g (Barrero et al. 2011). In China, wheat yield increase has largely depended on higher GPS and TKW (Zhang et al. 2012a). The frequencies of *Hap*-5A-1/2 and *Hap*-5A-4 rose steadily from 1971 to onwards during that time Chinese wheat varieties underwent significant increase in grain yield. This increase may also reflect continuous selection of TKW and GPS for higher grain yield in China (Fig. 9). Given that the favored haplotypes of *TaSnRK2.9*-5A were apparently selected in Chinese wheat breeding history, further selection of favored haplotypes might be helpful for continual improvement in wheat grain yield.

Wheat accessions from Pakistan and Europe were also used to evaluate *TaSnRK2.9*-5A in different geographical regions. In Pakistani accessions, although favored *Hap*-5A-1 (for TKW) was apparently selected in most areas from 1944 to 2016, the frequency of *Hap*-5A-4, the favored allele for GPS, remained very low, suggesting the potential of *Hap*-5A-4 introgression through FMs developed in this study (Fig. 6). The frequencies of favored haplotypes were higher in European population. *Hap*-5A-1/2 are more dominant in East European countries, while *Hap*-5A-4 is more dominant in West European countries, indicating further increase is still feasible in the regions where the favored haplotypes remained at lower frequencies, such as Finland and Germany (for *Hap*-5A-1) and Sweden, Switzerland, Hungary, Romania and Yugoslavia (for *Hap*-5A-4) (Fig. 7). The probable reason for the lower frequencies in these regions might be attributed to their breeding strategies deployed for gaining higher yields.

In genome-wide association studies for TKW and GPS, many loci were found to be linked with only one of the parameters. The superior haplotypes at these loci should increase the phenotypic value of one parameter without negatively affecting the phenotypic value of the others (Zhang et al. 2012a). Thus, selection of such QTL was likely a key reason in altering the association between TKW and GPS over time. From the results of this study, selection of favored haplotypes (*Hap*-5A-4) would also be helpful to increase GPS without reduction in mean TKW in wheat (Fig. 9). Genes and their functions in governing TKW are still mainly unidentified (Sajjad et al. 2017). Further selection of superior alleles might be useful for continued improvement in wheat.

High-throughput genotyping is of utmost importance for use in marker-assisted selection and genomic selection (Semagn et al. 2014). Gel-free KASP assays could significantly improve speed and efficiency of the selection in wheat breeding programs. Utilization of these multiple FMs will be more effective for yield improvement and can be instrumental in enhancing additive genetic variation (Wang et al. 2012). The gene identified here and molecular markers developed to identify haplotypes are useful for marker-assisted selection in breeding for TKW and GPS, which can be utilized alone or in combination with other functional markers.

### Author contribution statement

RJ, XM and SUR designed the experiments. SUR, JW, XC and XM performed the experiments. SUR analyzed the data. SUR, XM and RJ wrote the article. RJ and XM revised the article.

## Electronic supplementary material

Below is the link to the electronic supplementary material.

Supplementary material 1 (DOCX 145 kb)

Supplementary material 2 (DOCX 121 kb)

Supplementary material 3 (DOCX 179 kb)

Supplementary material 4 (DOCX 16 kb)
