# Supplementary material for: A wheat protein kinase gene TaSnRK2.9-5A associated with yield contributing traits
Source: Theor Appl Genet. 2018 Dec 5;132(4):907–19. doi: 10.1007/s00122-018-3247-7 (PMC6449320; doi:10.1007/s00122-018-3247-7)
Supplement: Supplementary file 1 — Supplementary material 1 (DOCX 145 kb) [file 122_2018_3247_MOESM1_ESM.docx]

*
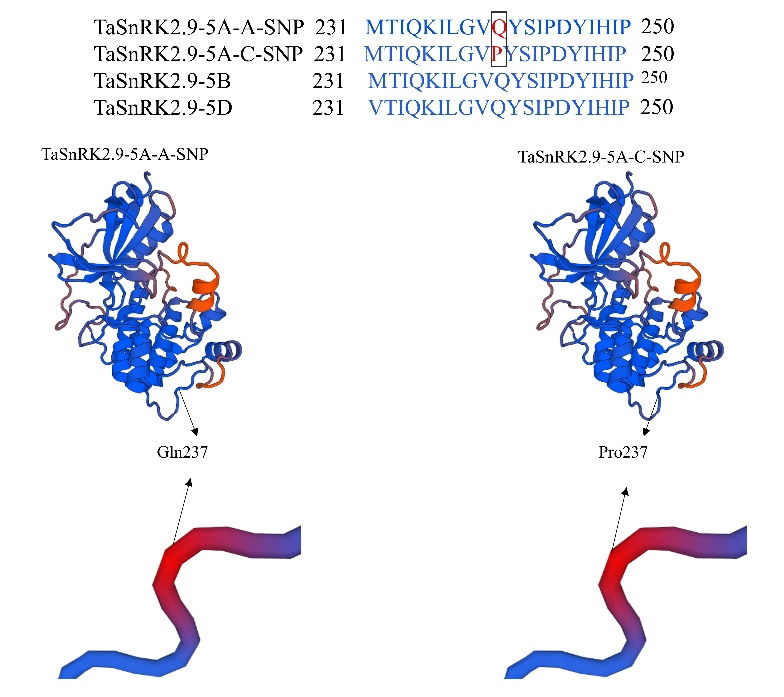
*

**Suplimentary Fig. 1 Secondary protein structure of TaSnRK2.9-5A**.

Common amino acid residues = blue font; rectangle = variation site
