# Supplementary figures and images for: A wheat protein kinase gene TaSnRK2.9-5A associated with yield contributing traits
Source: Theor Appl Genet. 2018 Dec 5;132(4):907–19. doi: 10.1007/s00122-018-3247-7 (PMC6449320; doi:10.1007/s00122-018-3247-7)

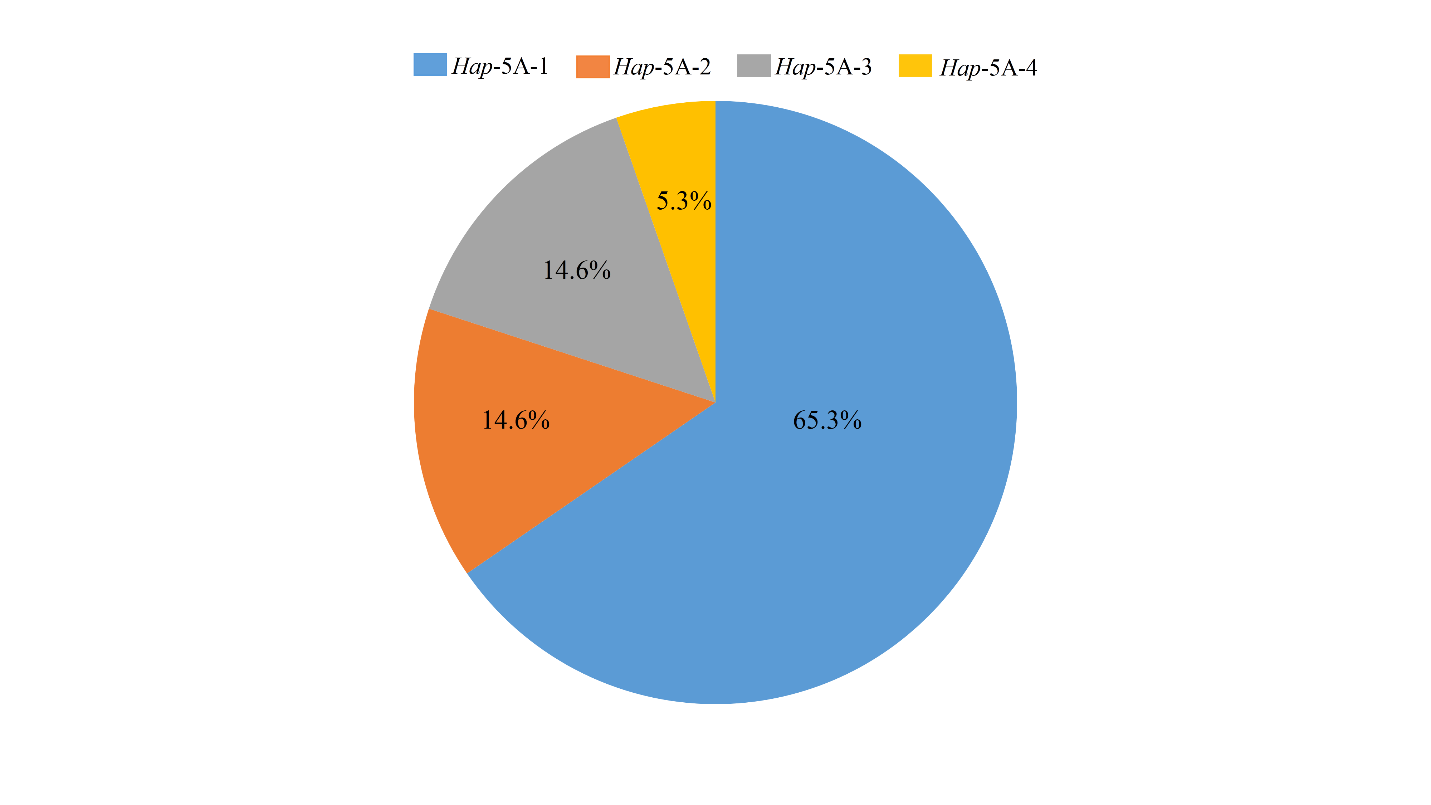


**Suplimentary Fig. 2** **Haplotype frequnecy in Pakistani accessions**

Supplement: Supplementary file 2 — Supplementary material 2 (DOCX 121 kb) [file 122_2018_3247_MOESM2_ESM.docx]

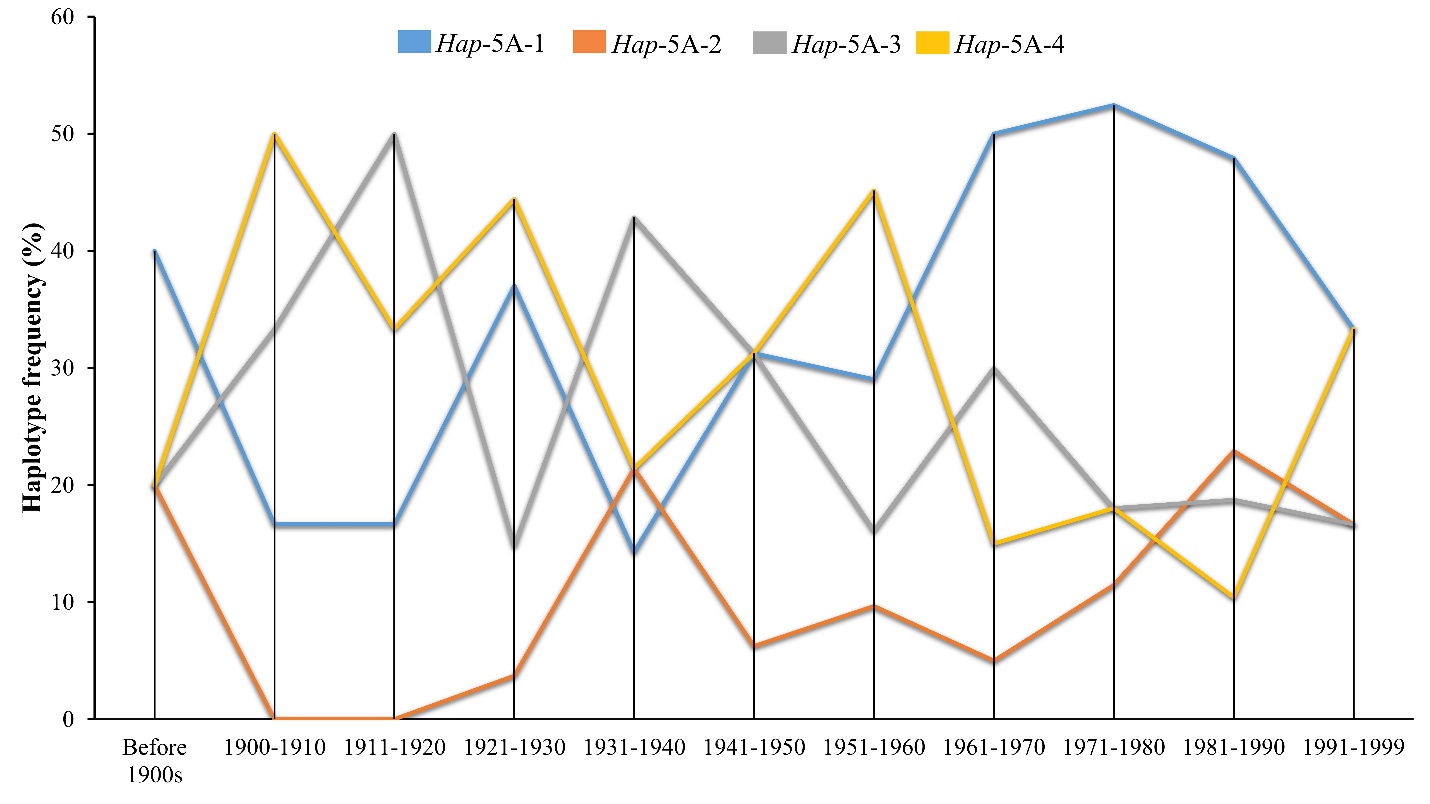


**Suplimentary Fig. 3** **Haplotype frequnecy over years in Europe**

Supplement: Supplementary file 3 — Supplementary material 3 (DOCX 179 kb) [file 122_2018_3247_MOESM3_ESM.docx]
