# Supplementary material for: A wheat protein kinase gene TaSnRK2.9-5A associated with yield contributing traits
Source: Theor Appl Genet. 2018 Dec 5;132(4):907–19. doi: 10.1007/s00122-018-3247-7 (PMC6449320; doi:10.1007/s00122-018-3247-7)
Supplement: Supplementary file 4 — Supplementary material 4 (DOCX 16 kb) [file 122_2018_3247_MOESM4_ESM.docx]

**Supplementary Table S1 water contents of different soil profiles for each growth season**

|  | Soil profile  (cm) | Booting stage | |  | Grain filling stage | |
| --- | --- | --- | --- | --- | --- | --- |
|  |  | Well-watered | Rainfed |  | Well-watered | Rainfed |
| 2015-Year | 20 | 18.6% | 9.8% |  | 12.3% | 7.8% |
|  | 40 | 13.9% | 12.1% |  | 9.4% | 5.4% |
|  | 60 | 14.2% | 10.3% |  | 8.8% | 6.0% |
|  | 80 | 16.3% | 10.8% |  | 12.8% | 8.2% |
|  | 100 | 15.8% | 12.4% |  | 16.4% | 9.7% |
|  | 120 | 19.3% | 11.4% |  | 16.6% | 13.0% |
|  | 140 | 19.9% | 14.0% |  | 17.5% | 11.5% |
|  | 160 | 21.2% | 16.5% |  | 17.1% | 14.6% |
|  |  |  |  |  |  |  |
| 2016-Year | 20 | 17.8% | 10.8% |  | 12.1% | 8.3% |
|  | 40 | 13.7% | 9.4% |  | 11.3% | 7.3% |
|  | 60 | 12.7% | 9.3% |  | 10.4% | 7.7% |
|  | 80 | 13.4% | 9.8% |  | 15.3% | 10.2% |
|  | 100 | 13.8% | 10.1% |  | 17.9% | 10.7% |
|  | 120 | 15.5% | 12.4% |  | 11.6% | 13.5% |
|  | 140 | 18.6% | 13.8% |  | 16.6% | 14.0% |
|  | 160 | 22.3% | 17.8% |  | 20.3% | 18.4% |
